# (1*RS*,6*SR*)-Ethyl 4,6-bis­(4-fluoro­phen­yl)-2-oxocyclo­hex-3-ene-1-carboxyl­ate

**DOI:** 10.1107/S1600536811000171

**Published:** 2011-01-12

**Authors:** Grzegorz Dutkiewicz, B. Narayana, K. Veena, H. S. Yathirajan, Maciej Kubicki

**Affiliations:** a Department of Chemistry, Adam Mickiewicz University, Grunwaldzka 6, 60-780 Poznań, Poland; b Department of Studies in Chemistry, Mangalore University, Mangalagangotri 574199, India; c Department of Studies in Chemistry, University of Mysore, Manasagangotri, Mysore 570 006, India

## Abstract

In the crystal structure of the title compound, C_21_H_18_F_2_O_3_, the cyclo­hexene ring has a slightly distorted sofa conformation; the two benzene rings are inclined by 76.27 (8)° and their planes make dihedral angles of 16.65 (10) and 67.53 (7)° with the approximately planar part of the cyclo­hexenone ring [maximum deviation 0.044 (2) Å, while the sixth atom is displaced by 0.648 (3) Å from this plane]. In the crystal, weak inter­molecular C—H⋯O, C—H⋯F and C—H⋯π inter­actions join mol­ecules into a three-dimensional structure.

## Related literature

For some biological applications of cyclo­hexa­nones, see: Li & Strobel (2001 ▶). For general properties, see: Jung (1991 ▶); Tabba *et al.* (1995 ▶). For asymmetry parameters, see: Duax & Norton (1975 ▶). For related structures, see: Anuradha *et al.* (2009 ▶); Li *et al.* (2009 ▶); Fun *et al.* (2008 ▶, 2009 ▶, 2010 ▶); Badshah *et al.* (2009 ▶).
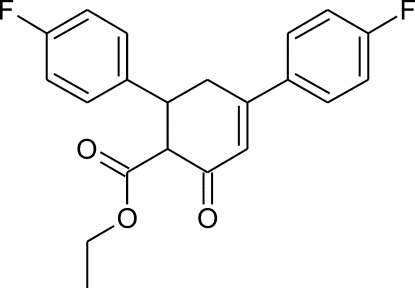


## Experimental

### 

#### Crystal data

C_21_H_18_F_2_O_3_
*M* *_r_* = 356.35
Monoclinic, 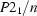

*a* = 11.062 (2) Å
*b* = 11.675 (3) Å
*c* = 13.854 (3) Å
β = 92.89 (2)°
*V* = 1787.0 (7) Å^3^
*Z* = 4
Mo *K*α radiation
μ = 0.10 mm^−1^
*T* = 295 K
0.45 × 0.2 × 0.2 mm

#### Data collection

Oxford Diffraction Xcalibur Sapphire2 diffractometer
Absorption correction: multi-scan (*CrysAlis PRO*; Oxford Diffraction, 2009 ▶) *T*_min_ = 0.947, *T*_max_ = 1.000
14582 measured reflections
3926 independent reflections
2590 reflections with *I* > 2σ(*I*)
*R*_int_ = 0.021

#### Refinement

*R*[*F* ^2^ > 2σ(*F* ^2^)] = 0.055
*wR*(*F* ^2^) = 0.168
*S* = 1.11
3926 reflections
235 parameters
H-atom parameters constrained
Δρ_max_ = 0.60 e Å^−3^
Δρ_min_ = −0.24 e Å^−3^

### 

Data collection: *CrysAlis PRO* (Oxford Diffraction, 2009 ▶); cell refinement: *CrysAlis PRO*; data reduction: *CrysAlis PRO*; program(s) used to solve structure: *SIR92* (Altomare *et al.*, 1993 ▶); program(s) used to refine structure: *SHELXL97* (Sheldrick, 2008 ▶); molecular graphics: *SHELXTL* (Sheldrick, 2008 ▶); software used to prepare material for publication: *SHELXL97*.

## Supplementary Material

Crystal structure: contains datablocks I, global. DOI: 10.1107/S1600536811000171/dn2648sup1.cif

Structure factors: contains datablocks I. DOI: 10.1107/S1600536811000171/dn2648Isup2.hkl

Additional supplementary materials:  crystallographic information; 3D view; checkCIF report

## Figures and Tables

**Table 1:** Hydrogen-bond geometry (Å, °)

*D*—H⋯*A*	*D*—H	H⋯*A*	*D*⋯*A*	*D*—H⋯*A*
C46—H46⋯O12^i^	0.93	2.56	3.244 (3)	130
C5—H52⋯F64^ii^	0.97	2.49	3.278 (2)	138
C5—H51⋯F44^iii^	0.97	2.54	3.484 (2)	165
C1—H1⋯*Cg*1^iv^	0.98	2.76	3.653 (3)	152

Symmetry codes: (i) 
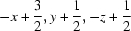
; (ii) 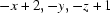; (iii) 
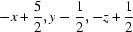
; (iv) 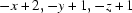.

**Fig. 1.**
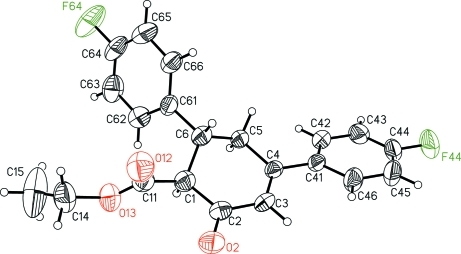
Anisotropic ellipsoid representation of the components of I together with atom labelling scheme. The ellipsoids are drawn at 50% probability level, hydrogen atoms are depicted as spheres with arbitrary radii.

**Fig. 2.**
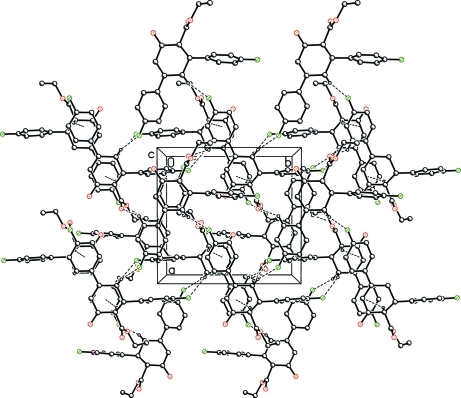
The crystal packing as seen along x-direction. Weak interactions (cf. text) are shown as dashed lines.

## supplementary crystallographic information

### Comment

An important feature of chalcones and their heteroanalogs is the ability to act as activated unsaturated systems in conjugated addition reactions of carbanions in the presence of basic catalysts ( Jung, 1991). This type of reaction may be exploited with the view of obtaining highly functionalized cyclohexene derivatives (Tabba *et al.*, 1995). Cyclohexenone derivatives possess a wide variety of biological activities, *e.g.* they were reported to have fungicidal and antitumor activities (Li & Strobel, 2001). Structures of some similar compounds have been reported earlier (for instance, ethyl 6-(4-chlorophenyl)-4-(4-methoxyphenyl)- 2-oxocyclohex-3-ene-1- carboxylate, Fun *et al.*, 2009, ethyl 4-(4-methoxyphenyl)-2-oxo-6- phenylcyclohex-3-ene-1-carboxylate, Fun *et al.*, 2008, ethyl 4-(4-bromophenyl)-6-(4-ethoxyphenyl)- 2-oxocyclohex-3-enecarboxylate, Badshah *et al.*, 2009, ethyl 6 - r-(2-chlorophenyl)-2-oxo-4- phenylcyclohex-3-ene-1 - t-carboxylate (Anuradha *et al.*, 2009). In the course of our studies on chalcone derivatives, we have synthesized some cyclohexene derivatives. here we report the crystal structure of (1RS,6SR) ethyl 4,6-bis(4-fluorophenyl)-2-oxocyclohex-3-ene-1-carboxylate (I, Scheme 1).

In I, the cyclohexene ring adopts slightly distorted sofa conformation (Fig. 1), the asymmetry parameter Δ*C_s_*^3^ (Duax & Norton, 1975) is 7.8°. This is also confirmed by least-squares calculations: five atoms C1 - C5 are almost coplanar, maximum deviation is 0.044 (2) Å, while the sixth atom, C6, is by 0.648 (3)Å out of this mean plane. The presence of two largest peaks at the difference Fourier map of *ca* 0.5 e.Å^-3^ (more than two times larger than the next peak) close to C1 and C6 atoms suggests the possibility of slight disorder of these two carbon atoms; this kind of disorder was observed previously in similar structures (*e.g.* Li *et al.*, 2009; Fun *et al.*, 2010)

The overall conformation of I (*cf.* Fig. 1) can be characterized by the dihedral angles between the phenyl rings, of 76.27 (8)°, and between these rings and the plane of cyclohexene ring which are equal to 16.65 (10)° for the ring at position 4 of the cyclohexene (*i.e.* next to the double bond) and 67.53 (7)° for fluorophenyl ring at position 6. In the crystal of the methyl analogue, methyl 4,6-bis(4-fluorophenyl)-2-oxocyclohex- 3-ene-1-carboxylate (Fun *et al.*, 2010) there are two symmetry independent molecules, but the overall conformation of both of them is similar to that of I. The dihedral angles between fluorophenyl rings are 79.7 (2)° and 73.7 (2)°, and the angles between the cyclohexene plane and the fluorophenyl rings at position 4 are 14.9° and 29.9°, while those at 6-postion: 73.7° and 84.0°. In the structure of ethyl 4-(4-bromophenyl)-6-(4-ethoxyphenyl)-2-oxo-4- phenylcyclohex-3-enecarboxylate (Badshah *et al.*, 2009) appropriate angles are 81.73 (12)°. 13.8 (3)° and 88.44 (17)°.

In the crystal structure the molecules are joined by weak C—H···O, C—H···F and C—H···πl interactions (Fig. 2).

### Experimental

A mixture of (2*E*)-1,3-bis(4-fluorophenyl)prop-2-en-1-one (0.01 mol) and ethyl acetoacetate (0.01 mol) were refluxed for 2 hr in 10–15 ml of ethanol in the presence of 0.8 ml 10% NaOH. The crystals were obtained by a slow evaporation from toluene solution. C_21_H_18_F_2_O_3,_ C: 70.71(70.78%); H: 5.07(5.09%); M.P-367 K.

### Refinement

Hydrogen atoms were located geometrically (*C*(methyl)-H 0.96 Å, C(CH2)—H 0.97 Å, C(CH)—H 0.98 Å, C(arom)-H 0.93 Å) and refined as a riding model; the *U*_iso_ values of H atoms were set at 1.2 (1.5 for CH_3_ group) times *U*_eq_ of their carrier atom.

### Crystal data

**Table:**

C_21_H_18_F_2_O_3_	*F*(000) = 744
*M**_r_* = 356.35	*D*_x_ = 1.325 Mg m^−^^3^
Monoclinic, *P*2_1_/*n*	Mo *K*α radiation, λ = 0.71073 Å
Hall symbol: -P 2yn	Cell parameters from 6918 reflections
*a* = 11.062 (2) Å	θ = 2.9–28.1°
*b* = 11.675 (3) Å	µ = 0.10 mm^−^^1^
*c* = 13.854 (3) Å	*T* = 295 K
β = 92.89 (2)°	Block, colourless
*V* = 1787.0 (7) Å^3^	0.45 × 0.2 × 0.2 mm
*Z* = 4	

### Data collection

**Table:**

Oxford Diffraction Xcalibur Sapphire2 diffractometer	3926 independent reflections
Radiation source: Enhance (Mo) X-ray Source	2590 reflections with *I* > 2σ(*I*)
graphite	*R*_int_ = 0.021
Detector resolution: 8.1929 pixels mm^-1^	θ_max_ = 28.2°, θ_min_ = 2.9°
ω–scan	*h* = −14→13
Absorption correction: multi-scan (*CrysAlis PRO*; Oxford Diffraction, 2009)	*k* = −15→14
*T*_min_ = 0.947, *T*_max_ = 1.000	*l* = −16→17
14582 measured reflections	

### Refinement

**Table:**

Refinement on *F*^2^	Primary atom site location: structure-invariant direct methods
Least-squares matrix: full	Secondary atom site location: difference Fourier map
*R*[*F*^2^ > 2σ(*F*^2^)] = 0.055	Hydrogen site location: inferred from neighbouring sites
*wR*(*F*^2^) = 0.168	H-atom parameters constrained
*S* = 1.11	*w* = 1/[σ^2^(*F*_o_^2^) + (0.0925*P*)^2^ + 0.1094*P*] where *P* = (*F*_o_^2^ + 2*F*_c_^2^)/3
3926 reflections	(Δ/σ)_max_ < 0.001
235 parameters	Δρ_max_ = 0.60 e Å^−^^3^
0 restraints	Δρ_min_ = −0.24 e Å^−^^3^

### Special details

**Table:**

Geometry. All e.s.d.'s (except the e.s.d. in the dihedral angle between two l.s. planes) are estimated using the full covariance matrix. The cell e.s.d.'s are taken into account individually in the estimation of e.s.d.'s in distances, angles and torsion angles; correlations between e.s.d.'s in cell parameters are only used when they are defined by crystal symmetry. An approximate (isotropic) treatment of cell e.s.d.'s is used for estimating e.s.d.'s involving l.s. planes.
Refinement. Refinement of *F*^2^ against ALL reflections. The weighted *R*-factor *wR* and goodness of fit *S* are based on *F*^2^, conventional *R*-factors *R* are based on *F*, with *F* set to zero for negative *F*^2^. The threshold expression of *F*^2^ > σ(*F*^2^) is used only for calculating *R*-factors(gt) *etc*. and is not relevant to the choice of reflections for refinement. *R*-factors based on *F*^2^ are statistically about twice as large as those based on *F*, and *R*- factors based on ALL data will be even larger.

### Fractional atomic coordinates and isotropic or equivalent isotropic displacement parameters (Å^2^)

**Table:**

	*x*	*y*	*z*	*U*_iso_*/*U*_eq_	
C1	0.74323 (18)	0.34723 (16)	0.44006 (15)	0.0454 (5)	
H1	0.7716	0.3445	0.5082	0.054*	
C11	0.61584 (18)	0.29820 (17)	0.43248 (16)	0.0454 (5)	
O12	0.55538 (16)	0.28780 (15)	0.35870 (12)	0.0687 (5)	
O13	0.58204 (14)	0.26686 (13)	0.51815 (11)	0.0580 (4)	
C14	0.4670 (2)	0.2089 (2)	0.5236 (2)	0.0717 (7)	
H142	0.4485	0.1662	0.4647	0.086*	
H141	0.4029	0.2642	0.5320	0.086*	
C15	0.4771 (4)	0.1311 (4)	0.6066 (3)	0.1367 (17)	
H153	0.4018	0.0914	0.6125	0.205*	
H152	0.4956	0.1743	0.6645	0.205*	
H151	0.5404	0.0765	0.5974	0.205*	
C2	0.74302 (18)	0.47139 (15)	0.40859 (14)	0.0419 (5)	
O2	0.65172 (14)	0.52929 (12)	0.41192 (11)	0.0585 (4)	
C3	0.85742 (17)	0.51786 (15)	0.37999 (14)	0.0405 (5)	
H3	0.8608	0.5956	0.3659	0.049*	
C4	0.95848 (16)	0.45610 (14)	0.37261 (12)	0.0338 (4)	
C41	1.07396 (16)	0.50657 (15)	0.34287 (12)	0.0360 (4)	
C42	1.18418 (18)	0.45216 (16)	0.36282 (14)	0.0431 (5)	
H42	1.1856	0.3826	0.3956	0.052*	
C43	1.2921 (2)	0.49924 (19)	0.33490 (16)	0.0525 (5)	
H43	1.3656	0.4629	0.3494	0.063*	
C44	1.2873 (2)	0.60004 (19)	0.28573 (15)	0.0533 (6)	
F44	1.39211 (13)	0.64622 (13)	0.25678 (11)	0.0824 (5)	
C45	1.1821 (2)	0.6560 (2)	0.26431 (16)	0.0609 (6)	
H45	1.1820	0.7250	0.2309	0.073*	
C46	1.0748 (2)	0.60919 (17)	0.29278 (15)	0.0508 (5)	
H46	1.0022	0.6471	0.2781	0.061*	
C5	0.95652 (16)	0.32986 (14)	0.39434 (14)	0.0381 (4)	
H52	0.9885	0.3175	0.4600	0.046*	
H51	1.0091	0.2906	0.3512	0.046*	
C6	0.82998 (18)	0.27777 (15)	0.38320 (15)	0.0440 (5)	
H6	0.8035	0.2847	0.3149	0.053*	
C61	0.83451 (16)	0.15033 (15)	0.40669 (15)	0.0417 (5)	
C62	0.8266 (2)	0.10661 (18)	0.49880 (16)	0.0557 (6)	
H62	0.8161	0.1562	0.5502	0.067*	
C63	0.8341 (2)	−0.0113 (2)	0.51561 (18)	0.0629 (6)	
H63	0.8284	−0.0411	0.5775	0.075*	
C64	0.8499 (2)	−0.08081 (17)	0.4390 (2)	0.0567 (6)	
F64	0.85470 (16)	−0.19620 (11)	0.45443 (14)	0.0970 (6)	
C65	0.8613 (2)	−0.04147 (17)	0.34810 (19)	0.0593 (6)	
H65	0.8746	−0.0914	0.2974	0.071*	
C66	0.85233 (19)	0.07498 (16)	0.33294 (16)	0.0491 (5)	
H66	0.8586	0.1033	0.2707	0.059*	

### Atomic displacement parameters (Å^2^)

**Table:**

	*U*^11^	*U*^22^	*U*^33^	*U*^12^	*U*^13^	*U*^23^
C1	0.0443 (11)	0.0402 (11)	0.0518 (12)	0.0010 (8)	0.0040 (9)	0.0007 (9)
C11	0.0393 (11)	0.0390 (10)	0.0576 (14)	0.0033 (8)	0.0016 (10)	−0.0015 (9)
O12	0.0698 (12)	0.0782 (12)	0.0573 (10)	−0.0158 (9)	−0.0052 (9)	−0.0040 (8)
O13	0.0470 (9)	0.0678 (10)	0.0592 (10)	−0.0111 (7)	0.0014 (7)	0.0059 (8)
C14	0.0485 (14)	0.0813 (17)	0.0861 (18)	−0.0126 (12)	0.0113 (13)	0.0076 (14)
C15	0.122 (3)	0.164 (4)	0.122 (3)	−0.078 (3)	−0.019 (2)	0.057 (3)
C2	0.0448 (11)	0.0346 (10)	0.0462 (11)	0.0060 (8)	0.0004 (9)	−0.0021 (8)
O2	0.0509 (9)	0.0459 (8)	0.0795 (11)	0.0148 (7)	0.0093 (8)	0.0028 (7)
C3	0.0488 (12)	0.0242 (8)	0.0483 (11)	0.0018 (8)	0.0000 (9)	0.0004 (8)
C4	0.0424 (10)	0.0291 (9)	0.0297 (9)	−0.0012 (7)	0.0005 (7)	−0.0009 (7)
C41	0.0451 (11)	0.0296 (9)	0.0334 (10)	−0.0040 (8)	0.0028 (8)	−0.0023 (7)
C42	0.0467 (11)	0.0351 (9)	0.0478 (12)	−0.0016 (8)	0.0057 (9)	−0.0003 (8)
C43	0.0462 (12)	0.0543 (12)	0.0578 (13)	−0.0023 (10)	0.0113 (10)	−0.0058 (11)
C44	0.0573 (14)	0.0563 (13)	0.0480 (12)	−0.0163 (11)	0.0189 (10)	−0.0014 (10)
F44	0.0719 (10)	0.0901 (11)	0.0885 (10)	−0.0272 (8)	0.0355 (8)	0.0064 (8)
C45	0.0766 (17)	0.0492 (12)	0.0581 (14)	−0.0137 (12)	0.0132 (12)	0.0146 (11)
C46	0.0587 (13)	0.0440 (11)	0.0499 (12)	−0.0008 (9)	0.0046 (10)	0.0142 (9)
C5	0.0398 (10)	0.0301 (9)	0.0450 (11)	0.0022 (7)	0.0073 (8)	0.0018 (8)
C6	0.0482 (12)	0.0328 (10)	0.0512 (12)	0.0010 (8)	0.0048 (9)	0.0011 (8)
C61	0.0360 (10)	0.0313 (9)	0.0581 (13)	−0.0032 (7)	0.0061 (9)	0.0024 (9)
C62	0.0621 (14)	0.0476 (12)	0.0579 (14)	−0.0016 (10)	0.0069 (11)	−0.0028 (10)
C63	0.0655 (16)	0.0566 (14)	0.0661 (16)	−0.0028 (11)	−0.0015 (11)	0.0240 (12)
C64	0.0511 (13)	0.0287 (10)	0.0884 (18)	−0.0007 (9)	−0.0142 (11)	0.0060 (11)
F64	0.1057 (13)	0.0316 (7)	0.1500 (15)	0.0013 (7)	−0.0293 (11)	0.0191 (8)
C65	0.0605 (15)	0.0387 (11)	0.0777 (17)	0.0028 (10)	−0.0064 (12)	−0.0118 (11)
C66	0.0511 (12)	0.0362 (10)	0.0597 (13)	−0.0016 (9)	0.0014 (10)	−0.0003 (9)

### Geometric parameters (Å, °)

**Table:**

C1—C6	1.508 (3)	C43—C44	1.359 (3)
C1—C2	1.514 (3)	C43—H43	0.9300
C1—C11	1.520 (3)	C44—C45	1.354 (3)
C1—H1	0.9800	C44—F44	1.358 (2)
C11—O12	1.199 (2)	C45—C46	1.382 (3)
C11—O13	1.314 (2)	C45—H45	0.9300
O13—C14	1.446 (3)	C46—H46	0.9300
C14—C15	1.466 (4)	C5—C6	1.527 (3)
C14—H142	0.9700	C5—H52	0.9700
C14—H141	0.9700	C5—H51	0.9700
C15—H153	0.9600	C6—C61	1.523 (2)
C15—H152	0.9600	C6—H6	0.9800
C15—H151	0.9600	C61—C66	1.370 (3)
C2—O2	1.218 (2)	C61—C62	1.381 (3)
C2—C3	1.450 (3)	C62—C63	1.398 (3)
C3—C4	1.339 (3)	C62—H62	0.9300
C3—H3	0.9300	C63—C64	1.354 (3)
C4—C41	1.484 (2)	C63—H63	0.9300
C4—C5	1.505 (2)	C64—C65	1.352 (3)
C41—C46	1.385 (3)	C64—F64	1.365 (2)
C41—C42	1.390 (3)	C65—C66	1.379 (3)
C42—C43	1.387 (3)	C65—H65	0.9300
C42—H42	0.9300	C66—H66	0.9300
			
C6—C1—C2	110.86 (16)	C45—C44—F44	118.8 (2)
C6—C1—C11	111.93 (17)	C45—C44—C43	122.6 (2)
C2—C1—C11	110.66 (16)	F44—C44—C43	118.6 (2)
C6—C1—H1	107.7	C44—C45—C46	119.2 (2)
C2—C1—H1	107.7	C44—C45—H45	120.4
C11—C1—H1	107.7	C46—C45—H45	120.4
O12—C11—O13	124.74 (19)	C45—C46—C41	120.8 (2)
O12—C11—C1	124.9 (2)	C45—C46—H46	119.6
O13—C11—C1	110.33 (18)	C41—C46—H46	119.6
C11—O13—C14	117.95 (18)	C4—C5—C6	113.10 (15)
O13—C14—C15	107.4 (2)	C4—C5—H52	109.0
O13—C14—H142	110.2	C6—C5—H52	109.0
C15—C14—H142	110.2	C4—C5—H51	109.0
O13—C14—H141	110.2	C6—C5—H51	109.0
C15—C14—H141	110.2	H52—C5—H51	107.8
H142—C14—H141	108.5	C1—C6—C61	115.37 (16)
C14—C15—H153	109.5	C1—C6—C5	109.70 (16)
C14—C15—H152	109.5	C61—C6—C5	110.32 (15)
H153—C15—H152	109.5	C1—C6—H6	107.0
C14—C15—H151	109.5	C61—C6—H6	107.0
H153—C15—H151	109.5	C5—C6—H6	107.0
H152—C15—H151	109.5	C66—C61—C62	118.03 (18)
O2—C2—C3	122.63 (17)	C66—C61—C6	118.18 (18)
O2—C2—C1	120.65 (17)	C62—C61—C6	123.74 (18)
C3—C2—C1	116.63 (16)	C61—C62—C63	120.8 (2)
C4—C3—C2	124.26 (17)	C61—C62—H62	119.6
C4—C3—H3	117.9	C63—C62—H62	119.6
C2—C3—H3	117.9	C64—C63—C62	117.9 (2)
C3—C4—C41	122.77 (16)	C64—C63—H63	121.0
C3—C4—C5	119.46 (16)	C62—C63—H63	121.0
C41—C4—C5	117.76 (15)	C65—C64—C63	123.24 (19)
C46—C41—C42	117.85 (17)	C65—C64—F64	118.5 (2)
C46—C41—C4	120.67 (17)	C63—C64—F64	118.3 (2)
C42—C41—C4	121.47 (16)	C64—C65—C66	117.9 (2)
C43—C42—C41	121.47 (19)	C64—C65—H65	121.1
C43—C42—H42	119.3	C66—C65—H65	121.1
C41—C42—H42	119.3	C61—C66—C65	122.1 (2)
C44—C43—C42	118.0 (2)	C61—C66—H66	118.9
C44—C43—H43	121.0	C65—C66—H66	118.9
C42—C43—H43	121.0		
			
C6—C1—C11—O12	−60.8 (3)	C44—C45—C46—C41	0.2 (3)
C2—C1—C11—O12	63.4 (3)	C42—C41—C46—C45	−0.4 (3)
C6—C1—C11—O13	118.10 (19)	C4—C41—C46—C45	−179.49 (18)
C2—C1—C11—O13	−117.69 (18)	C3—C4—C5—C6	23.3 (2)
O12—C11—O13—C14	4.2 (3)	C41—C4—C5—C6	−156.25 (16)
C1—C11—O13—C14	−174.69 (18)	C2—C1—C6—C61	−178.09 (16)
C11—O13—C14—C15	149.6 (3)	C11—C1—C6—C61	−54.0 (2)
C6—C1—C2—O2	148.72 (19)	C2—C1—C6—C5	56.6 (2)
C11—C1—C2—O2	23.9 (3)	C11—C1—C6—C5	−179.31 (16)
C6—C1—C2—C3	−34.5 (2)	C4—C5—C6—C1	−51.6 (2)
C11—C1—C2—C3	−159.36 (17)	C4—C5—C6—C61	−179.72 (15)
O2—C2—C3—C4	−177.82 (19)	C1—C6—C61—C66	144.90 (19)
C1—C2—C3—C4	5.5 (3)	C5—C6—C61—C66	−90.1 (2)
C2—C3—C4—C41	179.85 (16)	C1—C6—C61—C62	−37.8 (3)
C2—C3—C4—C5	0.3 (3)	C5—C6—C61—C62	87.2 (2)
C3—C4—C41—C46	−20.5 (3)	C66—C61—C62—C63	−1.3 (3)
C5—C4—C41—C46	159.02 (18)	C6—C61—C62—C63	−178.6 (2)
C3—C4—C41—C42	160.47 (18)	C61—C62—C63—C64	0.3 (3)
C5—C4—C41—C42	−20.0 (2)	C62—C63—C64—C65	1.4 (3)
C46—C41—C42—C43	0.9 (3)	C62—C63—C64—F64	−178.6 (2)
C4—C41—C42—C43	179.90 (17)	C63—C64—C65—C66	−2.1 (4)
C41—C42—C43—C44	−1.0 (3)	F64—C64—C65—C66	177.94 (19)
C42—C43—C44—C45	0.8 (3)	C62—C61—C66—C65	0.6 (3)
C42—C43—C44—F44	−179.08 (18)	C6—C61—C66—C65	178.04 (19)
F44—C44—C45—C46	179.47 (18)	C64—C65—C66—C61	1.0 (3)
C43—C44—C45—C46	−0.4 (4)		

### Hydrogen-bond geometry (Å, °)

**Table:**

*D*—H···*A*	*D*—H	H···*A*	*D*···*A*	*D*—H···*A*
C46—H46···O12^i^	0.93	2.56	3.244 (3)	130
C5—H52···F64^ii^	0.97	2.49	3.278 (2)	138
C5—H51···F44^iii^	0.97	2.54	3.484 (2)	165
C1—H1···Cg1^iv^	0.98	2.76	3.653 (3)	152

Symmetry codes: (i) −*x*+3/2, *y*+1/2, −*z*+1/2; (ii) −*x*+2, −*y*, −*z*+1; (iii) −*x*+5/2, *y*−1/2, −*z*+1/2; (iv) −*x*+2, −*y*+1, −*z*+1.
